# Spatiotemporal observation of light propagation in a three-dimensional scattering medium

**DOI:** 10.1038/s41598-021-01124-6

**Published:** 2021-11-08

**Authors:** Tomoyoshi Inoue, Yuasa Junpei, Seiya Itoh, Tatsuya Okuda, Akinori Funahashi, Tetsuya Takimoto, Takashi Kakue, Kenzo Nishio, Osamu Matoba, Yasuhiro Awatsuji

**Affiliations:** 1 Graduate School of Science and Technology, Kyoto Institute of Technology, Matsugasaki, Sakyo-ku, Kyoto, 606-8585 Japan; 2 Japan Society for the Promotion of Science, Kojimachi Business Center Building, 5-3-1 Kojimachi, Chiyoda-ku, Tokyo, 102-0083 Japan; 3 Graduate School of Engineering, Chiba University, 1-33, Yayoi-cho, Inage-ku, Chiba, 263-8522 Japan; 4 Advanced Technology Center, Kyoto Institute of Technology, Matsugasaki, Sakyo-ku, Kyoto, 606-8585 Japan; 5 Organization for Advanced and Integrated Research, Kobe University, Rokkodai 1-1, Nada, Kobe, 657-850 Japan; 6 Faculty of Electrical Engineering and Electronics, Kyoto Institute of Technology, Matsugasaki, Sakyo-ku, Kyoto, 606-8585 Japan

**Keywords:** Imaging and sensing, Microscopy

## Abstract

Spatiotemporal information about light pulse propagation obtained with femtosecond temporal resolution plays an important role in understanding transient phenomena and light–matter interactions. Although ultrafast optical imaging techniques have been developed, it is still difficult to capture light pulse propagation spatiotemporally. Furthermore, imaging through a three-dimensional (3-D) scattering medium is a longstanding challenge due to the optical scattering caused by the interaction between light pulse and a 3-D scattering medium. Here, we propose a technique for ultrafast optical imaging of light pulses propagating inside a 3D scattering medium. We record an image of the light pulse propagation using the ultrashort light pulse even when the interaction between light pulse and a 3-D scattering medium causes the optical scattering. We demonstrated our proposed technique by recording converging, refracted, and diffracted propagating light for 59 ps with femtosecond temporal resolution.

## Introduction

Optical imaging techniques are widely used to investigate the mechanisms underlying various physical, chemical, and biological phenomena^1,2^. Recently, real-time imaging of light pulse propagation has become an important topic for revealing previously unknown phenomena and properties that have not been observed before. Observing light pulse propagation with ultrahigh temporal resolution has provided novel insights in many scientific fields^3–5^. In particular, capturing the light pulse propagation inside a three-dimensional (3-D) scattering medium, which is closely related to the optical scattering properties, is desirable for establishing techniques for focusing light in deep tissue for high-resolution imaging and precision laser therapy^6–8^. In the past few decades, various techniques have been developed for observing the propagation behaviour of light pulses^9–18^. Although the performance of these techniques has been increasing, real-time observation of light pulse propagation inside such scattering media is still a long-standing challenge.

Light-in-flight recording by holography (LIF holography) is a powerful tool for observing a propagating light pulse in the form of a motion picture^19–23^. LIF holography can, in principle, record a motion picture of the light pulse propagation with a single shot because the technique does not require repetitive ultrashort light pulses. In particular, LIF holography can also provide a motion picture of light pulse propagation inside a 3-D scattering medium^21^. Digital light-in-flight recording by holography (DLIF holography)^24–28^ was also developed for observing light pulse propagation. DLIF holography is implemented by combining digital holography^29,30^ and LIF holography. Even though the technique, which allows the observation of light pulse propagation as a motion picture with a single-shot, has been shown to perform well, observing light pulse propagation in a 3-D scattering medium by DLIF holography has not been reported yet.

Here we present the observation of light pulse propagation inside a 3-D scattering medium by DLIF holography. We were able to experimentally confirm for the first time that the reconstructed images correspond to the light pulse itself and not that of the image of light pulse scattering surface. Introducing the digital recording process to the observation of the light pulse propagation is a promising investigation strategy to reveal the optimization condition of deep optical tissue imaging with an ultrashort light pulse. The results presented here are a significant step forward in revealing ultrafast phenomena related to light pulse propagation inside 3-D scattering media.

### Digital light-in-flight recording by holography (DLIF holography)

**Figure 1:**
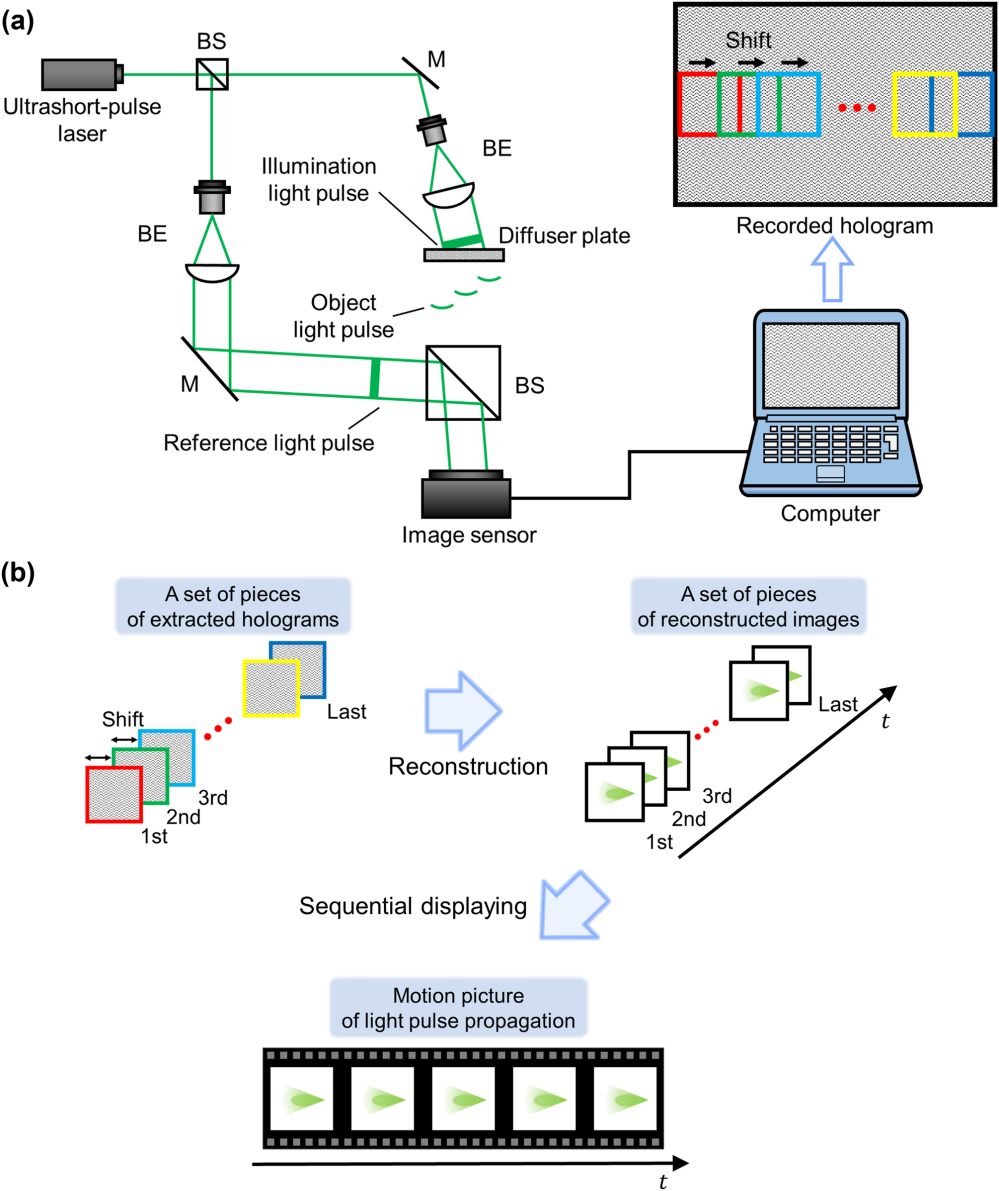
Schematic diagrams of the basic setups for DLIF holography. (**a**) Set-up for recording. BS, beam splitter; M, mirror; BE, beam expander. (**b**) Set-up for reconstruction. Figure 1 was originally created by the authors with PowerPoint for Microsoft 365 (https://www.microsoft.com/en-us/microsoft-365/powerpoint).

Figure 1a shows the top view of basic recording arrangement of DLIF holography. An ultrashort light pulse generated by an ultrashort pulsed laser was divided into two pulses by a beam splitter. Each pulse was collimated by a beam expander. One collimated ultrashort light pulse is called as the illumination light pulse. The illumination light pulse was obliquely incident on a diffuser plate at a certain angle. The scattered or diffused light pulse, called as the object light pulse, irradiates the image sensor. The other collimated light pulse is called as the reference light pulse. The reference light pulse was also obliquely incident on the image sensor. Only when both the object light pulse and the reference light pulse arrived at the image sensor at the same time, interference fringes are formed by both light pulses and are recorded on the image sensor. Since the reference light pulse obliquely sweeps over the image sensor, the light pulse propagation on the diffuser plate at each point was recorded in a different part of the image sensor. In the principle of the technique, the imaging speed is up to trillions of frames per seconds (see “Methods”). The recordable time in DLIF holography was determined by the time taken for the reference light pulse to pass through the image sensor (see “Methods”). In the previous study, the recordable time in DLIF holography was less than a few hundred femtoseconds^27^. To observe 3-D images of the light pulse propagation in a large time window, we introduced a method for extending the recordable time by using a diffraction grating^26,28^ (see “Methods”). Figure 1b shows a schematic diagram of the reconstruction process of DLIF holography. In the reconstruction process, sub-holograms were extracted from a single recorded hologram by shifting a certain number of pixels along the lateral direction, as shown in Fig. 1a. The diffraction integral was applied to each extracted hologram. By sequentially displaying reconstructed images, we could observe a motion picture of the propagating light pulse on the diffuser plate.

**Figure 2:**
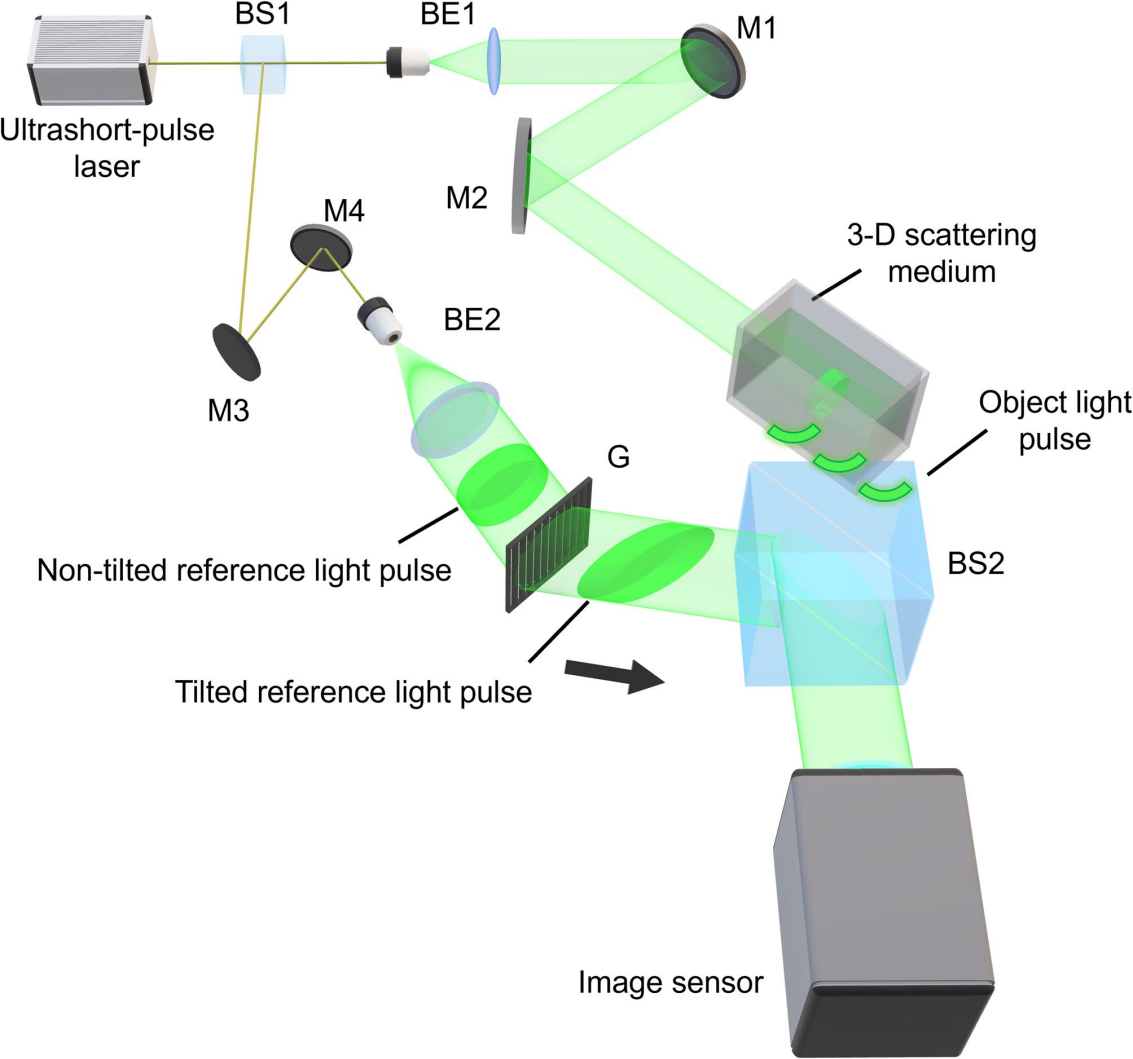
Schematic diagram of the experimental setup. BS1-2, beam splitters; BE1-2, beam expanders; M1-4, mirrors; G, grating. An ultrashort light pulse from an ultrashort pulsed laser was divided into two pulses by a beam splitter (BS1). Each light pulse was collimated by a beam expander. One light pulse was introduced into a transparent container filled with gelatin jelly. The container was set at an angle of 45° to the surface of an image sensor. The light pulse scattered from the gelatin jelly is called an object light pulse. The other collimated light pulse was introduced to the diffraction grating (G) to extend the recordable time of the motion picture. A light pulse diffracted from the diffraction grating was used as the tilted reference light pulse. The tilted reference light pulse was obliquely introduced to the image sensor. The two light pulses were recombined by another beam splitter (BS2). This figure was originally created by the authors with Blender 2.93.3 (https://www.blender.org/) and PowerPoint for Microsoft 365 (https://www.microsoft.com/en-us/microsoft-365/powerpoint).

Figure 2 shows a schematic diagram of the experimental setup for observing a light pulse propagating in the 3-D scattering medium (see “Methods”). The light pulse propagation in the 3-D scattering medium at each position was recorded in a different part of the image sensor. We could obtain a motion picture by sequentially displaying the reconstructed images in order as shown in Fig. 1b. We recorded the light pulse scattered from the gelatin jelly as an object light pulse. The light pulse scattered by the 3D scattering medium is scattered in all directions. Therefore, we define the light to be imaged as multiple scattering. We have observed the light pulse propagation characteristics such as (i) spatial focusing, (ii) refraction, and (iii) diffraction in the 3-D scattering medium.

## Results

### Experimental demonstration of the proposed technique

**Figure 3:**
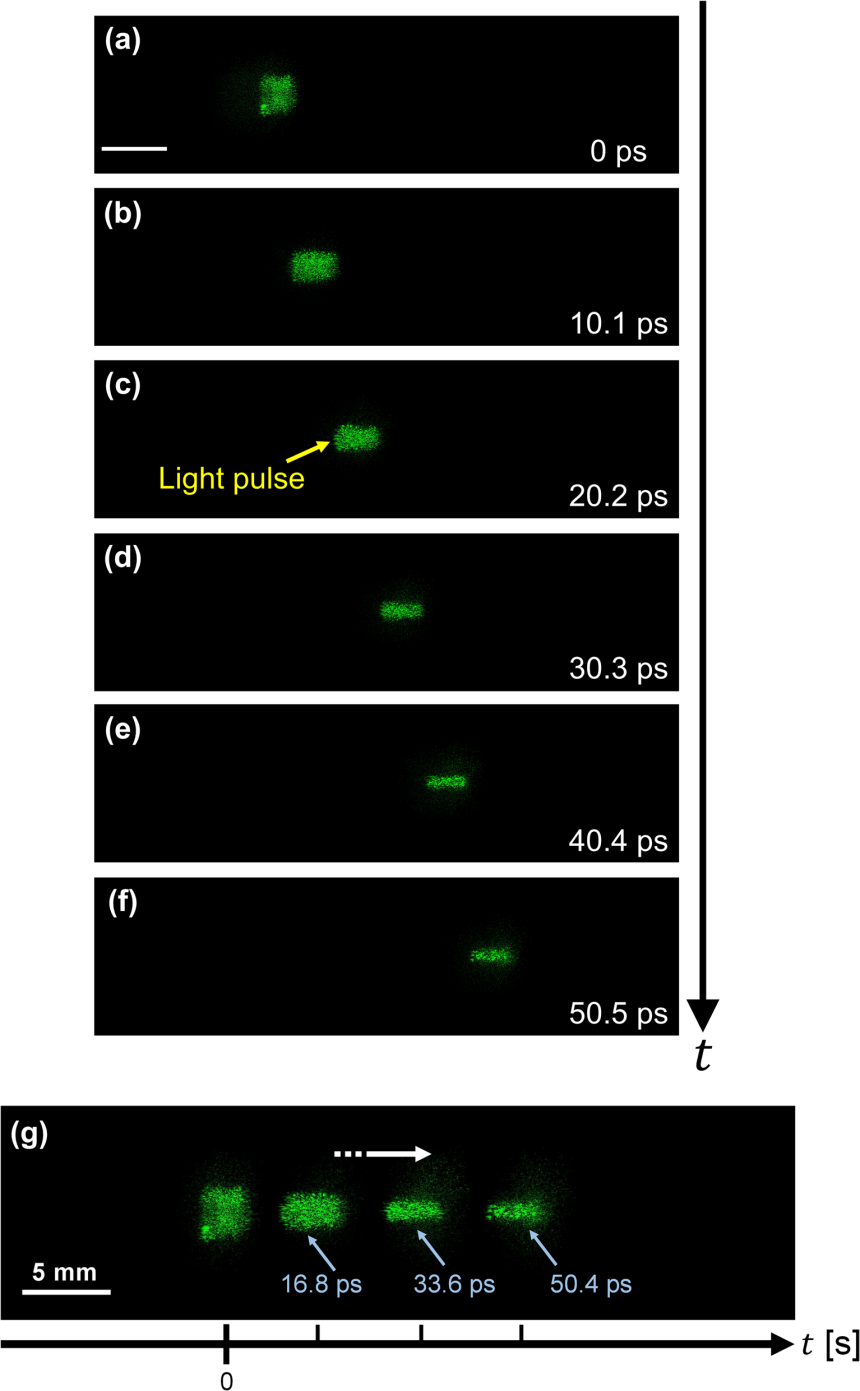
Frames extracted from the motion picture of the reconstructed images of a converging light pulse. (**a**–**f**), Converging light pulse in the 3-D scattering medium. The light pulse was propagating from left to right (see Video S1). The time interval between each picture was 10.1 ps. The light pulse was just focused in (**e**) and diverging in (**f**). Scale bar: 5 mm. (**g**) Figure shows the temporal change of the light pulse propagation by combining some reconstructed images. The striped arrow indicates the direction of the light pulse propagation.

First, to record the propagation of converging light pulse, a convex lens with a diameter of 4 cm and a focal length of 6 cm was placed just before the container. Figure 3 show the frames extracted from the motion picture. We reconstructed each image from 512 × 512 pixels extracted from the recorded hologram consisting of 4000 × 2624 pixels. The corresponding movie is Video S1. The actual duration of the motion picture was 59 ps, and the time interval between adjacent images was 10 ps. Sequence depth of Fig. 3 and the motion picture (Video S1) are 6 and 70, respectively. We determined the sequence depth (Number of frames) of Fig. 3 from the practical perspective (see “Methods”). We obtained this motion picture of the converging light pulse propagation by sequentially displaying the reconstructed images. The bright parts in Fig. 3 are the reconstructed images of the converging light pulse. The light pulse was converging and just focused, as shown in Fig. 3e. To show the exact timing and the temporal evolution of the reconstructed images, we combined and made some reconstructed images into Fig. 3g.

**Figure 4:**
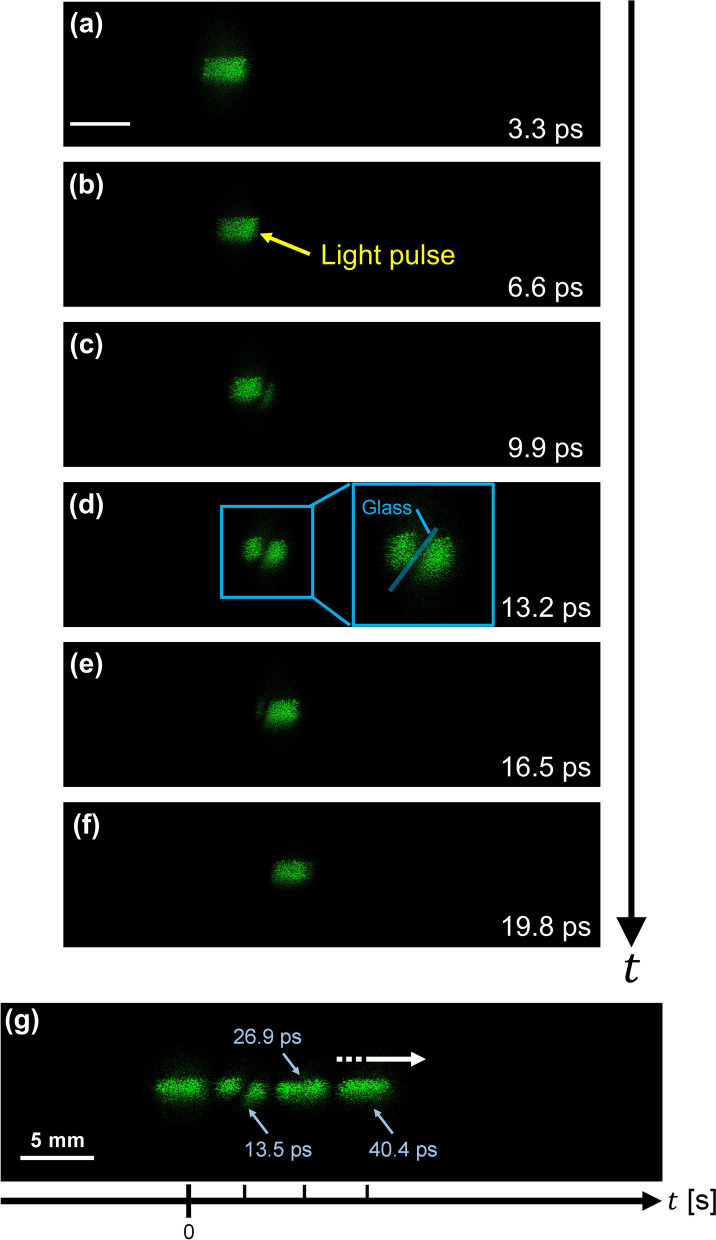
Frames extracted from the motion picture of the reconstructed images. (**a**–**f**), Propagating light pulse in the 3-D scattering medium. The light pulse was propagating from left to right (see Video S2). The time interval between each picture was 3.3 ps. The light pulse has not yet reached the glass in (**a**) and (**b**). The light pulse is entirely in the glass and refracted in (**c**–**e**). The light pulse has left the glass in (**f**). Scale bar: 5 mm. (**g**) Figure shows the temporal change of the light pulse propagation by combining some reconstructed images. The striped arrow indicates the direction of the light pulse propagation.

Second, we imaged a propagating light pulse being refracted in the 3-D scattering medium. Figure 4 show the frames extracted from the motion picture. We also reconstructed each image from 512 × 512 pixels extracted from the recorded hologram. The corresponding movie is Video S2. The actual duration of the motion picture was 59 ps, and the time interval between adjacent images in Fig. 4 was 3.3 ps. Sequence depth of Fig. 4 and the motion picture (Video S2) are 6 and 70, respectively. We have not shown all the frames but have chosen frames at certain intervals. We determined the sequence depth (number of the frames) of Fig. 4 from the practical perspective (see “Methods”). The bright spots in Fig. 4 indicate the reconstructed images of the light pulse. The light pulse in the 3-D medium was refracted at the medium-glass interface, as shown in Fig. 4c–e. To show the exact timing and the temporal evolution of the reconstructed images, we combined and made some reconstructed images into Fig. 4g. We observed that the light pulse changes direction as it crossed the boundary separating the medium and the glass, as shown in Fig. 4c–e,g.

**Figure 5:**
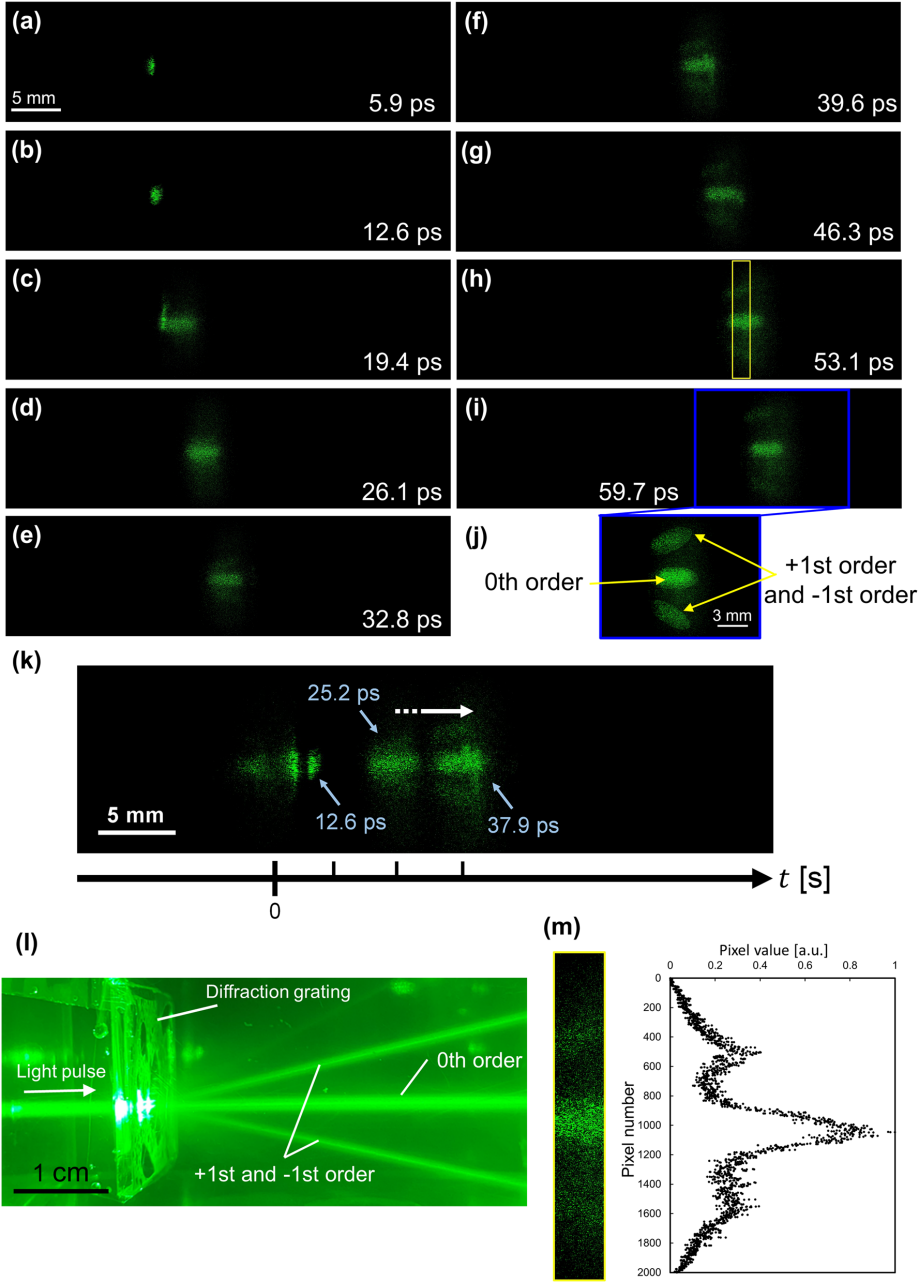
Observation of a light pulse being diffracted by the diffraction grating. The light pulse propagated in the 3-D scattering medium. (**a**–**i**), Frames extracted from left to right (see Video S3). The time interval between each picture was 6.7 ps. (**j**) Contrast-enhanced image of (**i**). (**k**) Figure shows the temporal change of the light pulse propagation by combining some reconstructed images. The striped arrow indicates the direction of the light pulse propagation. (**l**) Photograph taken by a conventional camera. (**m**) Normalized pixel values of the images of the diffracted light pulse. The measuring area corresponds to the yellow rectangle in (**h**). The number of pixels and the actual size of the measuring area were 300 (H) × 2000 (V) pixels and 1.77 (H) mm × 11.8 (V) mm, respectively.

Finally, we observed a propagating light pulse diffracted by a grating. We generated diffracted light pulses by using a phase grating. The spatial frequency of the grating was approximately 655 lines/mm, and the first-order diffraction angles were ±20° for 522 nm light. We also reconstructed each image from 512 × 512 pixels extracted from the recorded hologram. The actual duration of the motion picture was 59 ps, and the time interval between adjacent images was 6.7 ps. Sequence depth of the Fig. 5a–i and the motion picture (Video S3) are 9 and 70, respectively. We determined the sequence depth of Fig. 5 from the practical perspective (see “Methods”). The corresponding movie is Video S3. Figure 5a–i show the frames extracted from the motion picture of the reconstructed images. The incident light pulse was diffracted by the diffraction grating into the first orders, together with the zeroth order. Figure 5j shows a contrast-enhanced image of the two first-order diffraction beams and the zeroth-order diffraction beam. To show the exact timing and the temporal evolution of the reconstructed images, we combined and made some reconstructed images into Fig. 5k. Figure 5l shows a photograph of the light pulse being diffracted by the diffraction grating, acquired by a conventional camera. As seen from these results, we succeeded in observing the light pulse propagation in the 3-D scattering medium. We evaluated the diffracted light pulses by analysing the reconstructed image intensity. Figure 5m shows the normalized pixel value of the image of the diffracted light pulses. The measured area corresponds to the yellow rectangle in Fig. 5m. The number of pixels and the actual size of the measuring area were 300 (H) × 2000 (V) pixels and 1.77 mm (H) × 11.8 mm (V), respectively. Three peaks corresponding to the zero order and two first orders were confirmed as shown in Fig. 5m.

### Experimental evaluation of the image obtained by the proposed technique

In our demonstration, we presented a method to observe light pulse propagation inside a three-dimensional (3D) medium by using DLIF holography. The novelty of the study was that we were able to experimentally demonstrate that the captured image was that of the light pulse itself and not the image of light scattering surface inside the 3D scattering medium. This is the first time such a distinction has been reported in light pulse propagation measurement. In other words, the obtained image was not the image of an area where an illumination light pulse illuminates a diffuser plate, as reported in the previous study^27^. However, we could not determine whether the obtained images were a light pulse itself or the image of the area where the illumination light pulse illuminates the diffuser. To determine and confirm the image characteristics obtained in this study, we conducted a following experiment.

**Figure 6:**
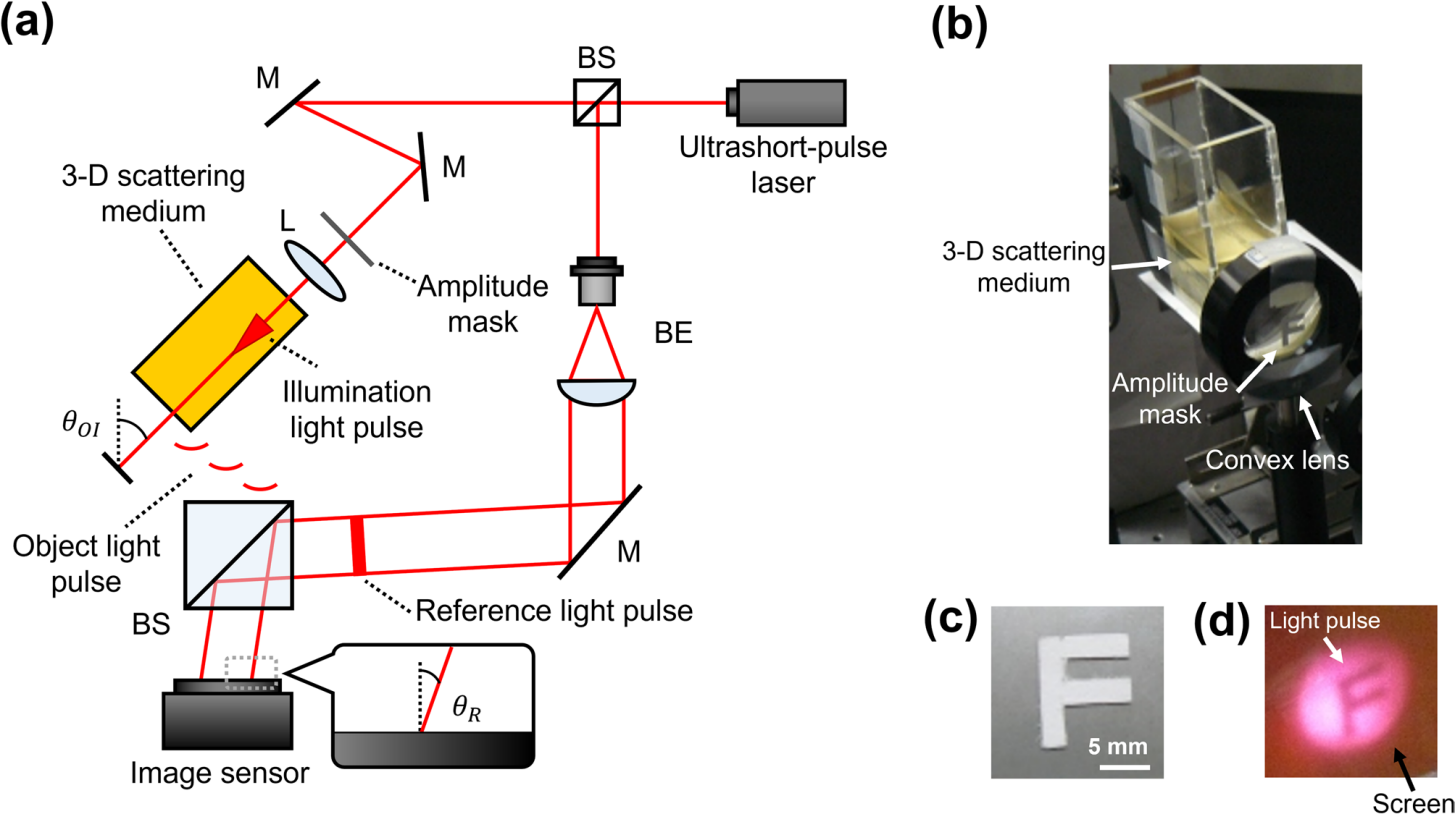
Experiment for evaluation of the reconstructed image. (**a**) Experimental setup. BS, beam splitter; M, mirror; BE, beam expander; L, convex lens. (**b**) Photograph of the 3D scattering media, convex lens, and Amplitude mask “F”. (**c**) Photograph of the amplitude mask. We chose and set “F” as an amplitude mask. The amplitude mask was attached. (**d**) The pulse surface of the illumination light pulse was spatially modulated by the letter “F”.

Figure 6a shows a schematic diagram of the experimental setup. The setup is almost the same as the one mentioned in the above (Fig. 2), but no grating for extending the recordable time is inserted in the optical path of the reference light pulse. To show that light pulse itself is being recorded and reconstructed, we created an amplitude mask on which the letter “F” was printed and displayed before a convex lens (Fig. 6b). Thus, when the size of the reconstructed image itself changes by the lens, it can be shown that the light pulse itself is being recorded. If the image of the area where the illumination light pulse and the 3D scattering medium overlap is recorded, a part of the light pulse and a part of the mask “F” are reconstructed in order. Also, considering the optical power loss caused by the amplitude mask, we used high power ultrashort pulsed laser, a mode-locked Ti:sapphire laser with a regenerative amplifier (Solstice, Spectra-Physics Inc.), to generate ultrashort light pulses. The center of wavelength and pulse duration of the ultrashort light pulses were 800 nm and 97 fs, respectively. The reference light pulse was obliquely incident to an image sensor with a certain incident angle. The incident angle of the reference light pulse was 10°. A transparent container filed with gelatin jelly was set to at an angle of 45° to the surface of an image sensor. The light pulse scattered from the gelatin jelly was used as an object light pulse. Figure 6b shows the placement of the 3D scattering medium and the amplitude mask. Figure 6c shows the detail of the amplitude mask. In order to record the converging light pulse inside the medium, a convex lens with a diameter of 40 mm and a focal length of 60 mm was placed just before the mask attached to the entrance face of the container. The pulse surface of the illumination light pulse was spatially modulated by the letter “F” as shown in Fig. 6d.

**Figure 7:**
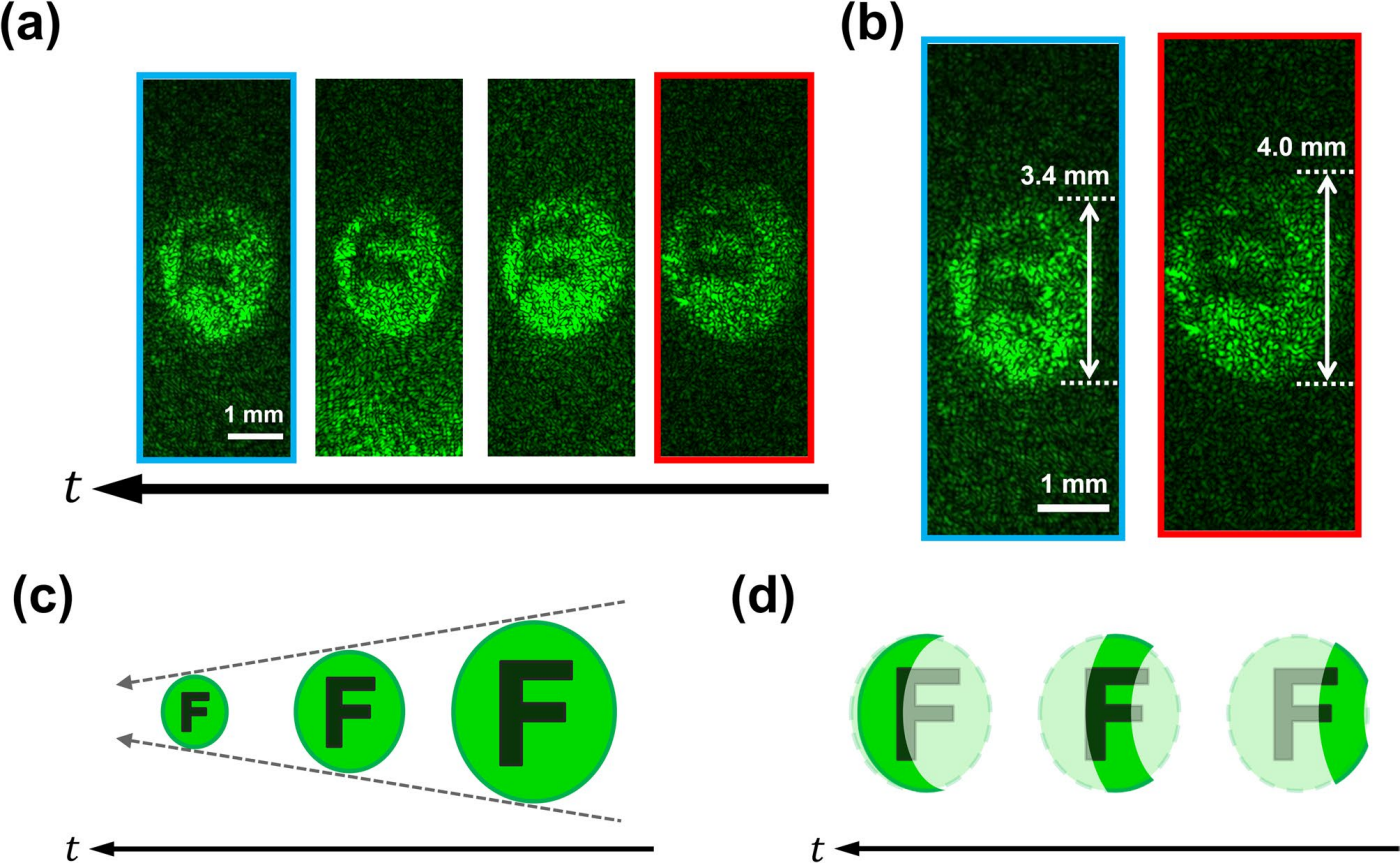
Experimental results and schematic illustration of the difference in experimental results. (**a**) Experimental results. The pulse surface was structured by the amplitude mask in which was printed the letter “F”. (**b**) Two frames extracted from the reconstructed images of the light pulse. (**c**) A schematic illustration of a behaviour of the reconstructed image when the light pulse itself was recorded. (**d**) A schematic illustration of a behaviour of the reconstructed image when the image of the area where the illumination light pulse illuminated the diffuser plate was recorded.

Figure 7 show experimental results and schematic of the schematic illustrations of the reconstructed images. Figure 7a shows the frames extracted from the experimentally obtained motion picture. We reconstructed each image from 512 × 512 pixels extracted from the recorded whole hologram consisting of 4000 × 2646 pixels. The actual time of the motion picture was 13.7 ps, and the time interval between the adjacent images in Fig. 7a was 3.8 ps. We also have not shown all the frames but have chosen frames at certain intervals. We can see the size of the reconstructed image of the light pulse itself was gradually decreasing as shown in Fig. 7b. In the other words, reconstructed image of the light pulse becomes smaller as it propagates (Fig. 7a). We explain the characteristics of the reconstructed images by schematic illustrations of the illumination light pulse spatially modulated by the letter “F”. Figure 7c shows a schematic illustration of a behaviour of the reconstructed image when the light pulse itself is recorded. Figure 7d shows a schematic illustration of a behaviour of the reconstructed image when the image of the area where the illumination light pulse illuminates the diffuser plate is recorded. When a 3D scattering medium was used (Fig. 3), only the size of the light pulse itself changes as shown in Fig. 7c. Thus, we are able to understand the whole image of the letter “F”. On the other hand, when the light pulse propagation was recorded using the diffuser plate, the image of the area where the light pulse intersects the diffuser plate was recorded and reconstructed as shown in Fig. 7d. Thus, a part of the amplitude mask “F” illuminated by the illumination light pulse is reconstructed, such as the image reported in the previous study^27^. Comparing the experimental results and these schematic illustrations, we can see the whole pattern of the amplitude mask instead of the part of the mask. This means that we were able to experimentally record and reconstruct the light pulse itself. Therefore, we succeeded in recording and observing the light pulse itself propagating inside a 3D scattering medium by using digital light-in-flight recording by holography.

## Discussion

The shape of the reconstructed image was longer in the horizontal direction than those recorded in the previous studies^27,28^. The light pulse scattered from each point of the 3D scattering medium propagated inside the medium. Thus, the light pulses scattered at different times were recorded simultaneously at the same area on the image sensor because the departing time of the object light pulse scattered from each portion of the medium differed depending on the recording geometry. As a result, when the extracted hologram was numerically processed to reconstruct the image, images of light pulses at different times were reconstructed all together. This means that the shape of the reconstructed light pulse differs from that of actual light pulse.

We have shown that light-in-flight imaging embodied by DLIF holography can be used to observe light pulse propagation inside a 3D scattering medium. Because of the ultrafast 3D and digital imaging capability, this imaging technique is one of the most powerful tools among many single-shot ultrafast optical imaging techniques^31^ and it can also be easily extended to observe the ultrafast dynamics of atoms and molecules when applied to electron holography or X-ray holography^32,33^. Our results will open many opportunities for revealing ultrafast light-matter interactions, not only in industrial science and engineering but also life science and biomedicine.

## Methods

### Experimental evaluation of the image obtained by the proposed technique

The recordable time^22,34^, $$ {t}_{rec}$$, is equal to the time required for the reference light pulse to cross the recording material laterally and can be written as follows:

1 $$t_{rec} = \frac{{L\sin \theta_{R} }}{c},$$

where $${c, L}$$ and $$ {\theta}_{R}$$ are the speed of light in air, the lateral length of the recording material, and the incident angle of the reference light pulse to the image sensor, respectively. The recordable time $$ {t}_{rec}$$ is mainly limited by the lateral size of the recording material.

To observe 3-D images of the light pulse propagation in a large time window, we introduce a method for extending the recordable time by using a diffraction grating^26^. In order to observe the light pulse propagation in a 3-D scattering medium, it is necessary to achieve a longer recordable time of a few tens of picoseconds. Propagation through optical components such as a grating or a prism causes different frequency components of the light pulse to propagate at different angles. The dispersion is called as an angle dispersion, which causes pulse front tilt^35^. For the grating, the pulse front is caused by the accumulation of optical path length differences between two neighboring lines of the grating. In this case, the reference light pulse was incident perpendicular to the grating and diffracted. Therefore, the pulse front of the diffracted light pulse is parallel to the diffraction grating surface. The tilted light pulse sweeps over the recording material more slowly than a non-tilted light pulse. This enables the recordable time of the motion picture to be extended. The recordable time by using the tilted light pulse, $$ {t}_{rectilt}$$, can be written as follows:

2 $$t_{rectilt} = \frac{{L\sin (\theta_{R} + \theta_{tilt} )}}{{c\cos \theta_{tilt} }},$$

where ${\theta}_{tilt}$ is the pulse front tilt angle. We used a tilted reference light pulse obtained by collimating the reference light pulse and introducing it to a diffraction grating.

### Experimental setup

A mode-locked Yb:YVO_4_ pulsed laser (HighQ-2 SHG, Spectra-Physics Inc.) was used for the light source. The center wavelength and the duration of the light pulses emitted from the laser were 522 nm and 178 fs, respectively. We used a digital CCD camera (OLCA-HR, Hamamatsu Photonics K. K.) to record the hologram. The number of pixels and the pixel pitch of the camera were 4000 (H) × 2624 (V) pixels and 5.9 μm × 5.9 μm, respectively. By using two-beam interference exposure, we fabricated a transmission diffraction grating on a holographic plate (Konica P-5600) for tilting the pulse front of the reference light pulse. The tilt angle of the pulse front was 40°.

In the hologram recording process, an ultrashort light pulse from an ultrashort pulsed laser was incident on a beam splitter. The light pulse was divided into two pulses, and each light pulse was collimated by a beam expander. The illumination light pulse was introduced into a transparent container filled with gelatin jelly. The container was set at an angle of 45° to the surface of an image sensor. The light pulse scattered from the gelatin jelly was called as the object light pulse. The other collimated light pulse was introduced to the diffraction grating in order to extend the recordable time of the motion picture obtained by DLIF holography. A light pulse diffracted from the diffraction grating was used as the tilted reference light pulse. The tilted reference light pulse was obliquely incident on the image sensor.

### Sequence depth and frame rate of digital light-in-flight recording by holography

We denote a sequence depth (Number of frames) of digital light-in-flight recording by holography. In the recording process, the pulse front of the reference light pulse intersects with different areas along the lateral direction of the image sensor because the reference light pulse is obliquely incident to the image sensor. And then, the interference fringe is formed by the superposition of reference light pulse and object light pulse and is recorded on the image sensor. In the principle of digital light-in-flight recording by holography, it is considered that a different image can be recorded when the pulse front of the reference light pulse moves laterally by one pixel. In other words, the light propagation is recorded by the number of frames corresponding to the number of pixels in the horizontal direction of the image sensor. Therefore, a maximum sequence depth of the technique is determined by the number of pixels in the lateral direction of the image sensor. We used a CCD camera (ORCA-HR, Hamamatsu Photonics K.K.) to record the hologram. The number of pixels of the camera is 4000 (H) × 2624 (V) pixels. Thus, the maximum sequence depth of the technique is 4000. In practice, we extract holograms (sub-holograms) from a recorded hologram by shifting a certain number of pixels along the lateral direction of the image sensor. The pixel size of the shift is commonly a several tens or hundreds of pixels on the image sensor. As a result, the total sequence depth of this study is lower than the maximum sequence depth.

We also denote a frame rate of digital light-in-flight recording by holography. The frame rate ${ \delta }_{t}$ is inversely proportional to the time required for the reference light pulse to cross the recording material laterally (Eq. 1). As described same above, it is considered that a different image can be recorded when the pulse front of the reference light pulse moves laterally by one pixel. Therefore, the frame rate ${ \delta }_{t}$ is given by

3 $$\delta_{t} = \frac{{N_{x} }}{{t_{rec} }} = \frac{{N_{x} c}}{{L\sin \theta_{R} }},$$

where $$ {N}_{x}$$, $$ c, L$$ and $$ {\theta}_{R}$$ are the number of pixels of the image sensor in lateral direction, the speed of the light in the air, the lateral size of the image sensor, and the incident angle of the reference light pulse, respectively. Similarly, we can determine a frame rate when the recordable time of the technique is extended by using the tilted light pulse. The frame rate can be expressed as

4 $$\delta_{tex} = \frac{{N_{x} }}{{t_{rectilt} }} = \frac{{N_{x} c\cos \theta_{tilt} }}{{L\sin (\theta_{R} + \theta_{tilt} )}},$$

where ${\theta}_{tilt}$ is the pulse front tilt angle. Then, ${{\delta}_{tex}}$ in this experiment was calculated from (4), as follow: ${ \delta }_{tex}$ = 58 × 10^12^ fps = 58 Tfps. This means that digital light-in-flight recording by holography achieves several tens trillion frames per second imaging.

## Supplementary Information

Supplementary Information 1.

Supplementary Video 1.

Supplementary Video 2.

Supplementary Video 3.
