# Supplementary material for: Spatiotemporal observation of light propagation in a three-dimensional scattering medium
Source: Sci Rep. 2021 Nov 8;11:21890. doi: 10.1038/s41598-021-01124-6 (PMC8576009; doi:10.1038/s41598-021-01124-6)
Supplement: Supplementary file 1 — Supplementary Information 1. [file 41598_2021_1124_MOESM1_ESM.pdf]

Supplementary Information: Spatiotemporal observation of light propagation in a three-dimensional scattering medium

Tomoyoshi Inoue[1,2], Yuasa Junpei[1], Seiya Itoh[1], Tatsuya Okuda[1], Akinori Funahashi[1], Tetsuya Takimoto[1], Takashi Kakue[3], Kenzo Nishio[4], Osamu Matoba[5], and Yasuhiro Awatsuji[6,*]

[1]Kyoto Institute of Technology, Graduate School of Science and Technology, Matsugasaki, Sakyo-ku, Kyoto, 606-8585, Japan

[2]Japan Society for the Promotion of Science, Kojimachi Business Center Building, 5-3-1 Kojimachi, Chiyoda-ku, Tokyo 102-0083, Japan

[3]Chiba University, Graduate School of Engineering, 1-33, Yayoi-cho, Inage-ku, Chiba 263-8522, Japan

[4]Kyoto Institute of Technology, Advanced Technology Center, Matsugasaki, Sakyo-ku, Kyoto, 606-8585, Japan

[5]Kobe University, Organization for Advanced and Integrated Research, Rokkodai 1-1, Nada, Kobe 657-850, Japan

[6]Kyoto Institute of Technology, Faculty of Electrical Engineering and Electronics, Matsugasaki, Sakyo-ku, Kyoto, 606-8585, Japan

*corresponding.author: awatsuji@kit.ac.jp

Movie Legends

Video S1.mp4

A converging light pulse in a 3D scattering medium.

A movie showing a converging light pulse in a 3D scattering medium. The light pulse was propagating from left to right. The light pulse was converging and focused. The actual duration of this motion picture was 59 ps.

Video S2. mp4

A propagating light pulse being refracted in a 3D scattering medium.

A movie showing a propagating light pulse being refracted in a 3D scattering medium. The light pulse was propagating from left to right. We can see that the light pulse changed direction as it crossed the boundary separating the medium and the glass. The actual duration of this motion picture was 59 ps.

Video S3.mp4

A light pulse being diffracted by a diffraction grating in a 3D scattering medium.

A movie showing a light pulse being diffracted by a diffraction grating in a 3D scattering medium. The light pulse was propagating from left to right. We can see that the incident light pulse was diffracted by the diffraction grating into the first orders and the zeroth order. The actual time of this motion picture was 59 ps.
